# Editorial Comment: Lessons learned after 20 years' experience with penile fracture

**DOI:** 10.1590/S1677-5538.IBJU.2019.0367.1

**Published:** 2020-02-20

**Authors:** Aderivaldo Cabral Dias, Homero Ribeiro

**Affiliations:** 1 Unidade de Urologia do Hospital de Base do Distrito Federal DF Brasil Unidade de Urologia do Hospital de Base do Distrito Federal, DF, Brasil; 2 Universidade Estadual de Campinas Faculdade de Ciências Médicas Departamento de Cirurgia CampinasSP Brasil Disciplina de Urologia, Departamento de Cirurgia, Faculdade de Ciências Médicas, Universidade Estadual de Campinas - UNICAMP, Campinas, SP, Brasil

## COMMENT

The article describes the authors' 20-year experience with penile fracture, gained from their work at largest emergency department of the city of Rio de Janeiro (1). This study follows previous publications on the subject of penile fracture by the same group of authors (2-4), and provides the practising urologist with valuable information about the diagnosis and treatment of the disease.

Although there is justifiable debate about whether one should surgically approach penile fractures through a subcoronal (the authors' - and ours' - choice), penoescrotal or perineal incision, the attending urologist must first determine whether the patient has, in fact, a penile fracture, or a vascular injury mimicking a penile fracture, i.e. a false penile fracture (5-7). The authors' no-nonsense approach to this diagnostic problem was to consider patients that did not describe immediate detumescence after injury, presenting small-to-moderate penile hematoma (or none at all) and without abnormalities in the palpation of the corpora cavernosa as having a lower likelihood of penile fracture - and offer to these patients supplemental imaging studies or conservative treatment with close follow-up. The reader must be cautioned, however, that such line of action is grounded by the authors' large experience, and that the default approach in clinically diagnosed penile fracture patients is surgical exploration.

Another facet of the authors' large experience with the disease can be observed in their approach to synchronous urethral injuries, diagnosed in 54 of their patients (18.7%). Preoperative retrograde urethrography (RUG) was performed in a little less than half of those (25/54, 47%), and their logic in restricting the indication of RUG is simple to follow: Urethral injuries are not rare in penile fracture; RUG has been associated with high false-negative rates (reference 14 in the article); penile degloving provides excellent exposure to the penile urethra; urethral repair can be easily performed concomitant with tunical repair; ergo, one can forgo RUG in these cases. Yet, one should not promptly waive RUG if the surgical plan involves an incision directly to the site of injury, even more so if there is any sign of urethral injury, e.g. urethral bleeding, urinary retention.

Our last comment addresses the fact that the study included 285 patients and 288 penile fractures, as 3 patients had a recurrent fracture - at the same site as the first one. This finding calls to attention that changes in the biomechanical properties of the tunica albuginea at the site of injury predosposes the organ to repeat fracture. The last consideration regards the patient's behavior during the sexual act. Since the persistence of the sexual behavior that caused the fracture may lead to a recurrent fracture, one can conjecture that the sparseness of recurrent fractures may stem from modifications in sexual behavior - decreases in the frequency of intercourse, avoidance of certain positions, for instance - likely associated with the already documented negative long-term psychological impact of the disease (8). Such modifications in sexual behavior, while detrimental to the patient's and partner's quality-of-life, may subliminally protect the patient against another frature. Perhaps one lesson to be learned from this is that penile fracture patients may benefit from more detailed information about the mechanism of fracture, as well as from sexual counseling.
